# Clinical characteristics of patients with metastatic castration-resistant prostate cancer after treatment with combined androgen blockade

**DOI:** 10.1186/s12894-023-01233-6

**Published:** 2023-04-28

**Authors:** Daisuke Obinata, Sho Hashimoto, Hideaki Uchida, Ken Nakahara, Tsuyoshi Yoshizawa, Junichi Mochida, Kenya Yamaguchi, Satoru Takahashi

**Affiliations:** Department of Urology, Nihon University School of Medicine, 30-1, Oyaguchikamicho, Itabashi-Ku, Tokyo, 173-8610 Japan

**Keywords:** CRPC, Anti-androgens, Prostate cancer

## Abstract

**Background:**

Although the second-generation androgen receptor inhibitors and taxanes have recently been recommended for the initial treatment of metastatic prostate cancer, bicalutamide and flutamide are still used in a large number of cases. Therefore, it is important to elucidate the clinical characteristics of these treated CRPC cases and their sensitivity to the currently used therapeutic agents. We aimed to examine the outcomes of metastatic castration-resistant prostate cancer following combined androgen blockade as initial therapy at our institution.

**Methods:**

Ninety-four patients who developed metastatic castration-resistant prostate cancer after hormonal treatment with combined nonsteroidal androgen receptor antagonists and continuous androgen deprivation therapy between January 2015 and December 2020 were included. The presence of visceral metastases, duration of efficacy of each treatment, and overall survival after castration-resistant prostate cancer were evaluated.

**Results:**

Patients with a longer duration of castration-resistant prostate cancer tended to have a longer response duration to subsequent enzalutamide administration (p = 0.003). Patients who achieved a 90% reduction in prostate-specific antigen levels with enzalutamide had a significantly better castration-resistant prostate cancer prognosis (p = 0.002). Meanwhile, those with visceral metastases at the time of castration-resistant prostate cancer diagnosis had a significantly poorer prognosis (p < 0.001). A positive correlation was observed between the treatment efficacy of abiraterone and taxanes for castration-resistant prostate cancer.

**Conclusion:**

The study provides scientific evidence to support that patients with longer time to castration-resistant prostate cancer are more sensitive to enzalutamide, and the use of abiraterone between docetaxel and cabazitaxel has favorable prognostic impact. These findings provide instrumental evidence that can enable better treatment selection for prostate cancer patients.

**Supplementary Information:**

The online version contains supplementary material available at 10.1186/s12894-023-01233-6.

## Introduction

The androgen receptor (AR) and its subsequent signaling pathways play important roles in the growth and progression of prostate cancer [1]. Therefore, drug therapy targeting the AR signaling pathway is a mainstay of treatment for metastatic prostate cancer. Androgen deprivation therapy (ADT) is a primary systemic treatment for advanced prostate cancer. Combined androgen blockade (CAB) therapy consists of testosterone suppression in combination with ADT and nonsteroidal AR antagonists (NSA), such as bicalutamide and flutamide. CAB is an effective treatment for advanced prostate cancer with metastases; however, it induces resistance and leads to a refractory cancer called castration-resistant prostate cancer (CRPC) [1]. However, the AR signaling pathway continues to be activated in early CRPCs, even in a low-androgen environment.

Second-generation AR inhibitors and the taxane-based anticancer agents docetaxel and cabazitaxel have demonstrated excellent progression-free survival in metastatic CRPC [2–4]. Additionally, these agents have recently been recommended for the initial treatment of metastatic prostate cancer [5, 6]. However, data from the National Database of Health Insurance Claims and Specific Health Checkups of Japan database show that bicalutamide and flutamide are still used for the treatment of a large proportion of metastatic prostate cancer cases, with bicalutamide and flutamide prescribed for 65,321,653 tablets per year in 2018 [7]. Therefore, it is important to elucidate the treatment modalities and clinical characteristics of CRPC after failure with standard therapy.

This study aimed to analyze the clinical features of metastatic CRPC following bicalutamide or flutamide failure.

## Materials and methods

In our institution, we treated 182 consecutive patients with metastatic CRPC between January 2015 and December 2020. Of these, we investigated 94 patients with metastatic CRPC who were diagnosed at our institution and treated with CAB and who had no history of female hormone therapy. CRPC was diagnosed as castration (testosterone < 50 ng/dL) with biochemical and/or clinical progression according to the PCWG2 and RECIST 1.1 criteria [8]. In addition to drug treatment with CAB, total prostatectomy was performed in 5 patients and radiation therapy in 12 patients. None of the patients previously received systemic anticancer therapy for advanced or metastatic disease. Initial TNM classification, Gleason scores, presence of visceral metastases, duration of efficacy of each treatment, time to CRPC from diagnosis, best prostate-specific antigen (PSA), alkaline phosphatase (ALP), and C-reactive protein (CRP) response in each CRPC treatment, and overall survival (OS) after CRPC were evaluated. When side effects or clinical progression were observed, changes in therapeutic agents were made at the discretion of the attending physician in accordance with the Japanese guidelines for prostate cancer treatment.

Continuous data were presented as mean ± standard deviation (SD) or median with minimum and maximum values. The Student’s t-test was used to compare continuous data between the groups. One-way ANOVA with the Turkey-Kramer test was used to compare continuous data among three groups. Chi-square and Fisher’s exact tests were used to analyze categorical variables. Multivariate linear regression models were used to evaluate whether time to CRPC was an independent predictor of the effect of each CRPC treatment. OS analyses were conducted using the Kaplan–Meier method, and survival characteristics were compared using the log-rank test. Univariate and multivariate analyses were performed with each drug as a variable to determine its correlation with OS. The correlation of the best PSA response (minimum PSA during treatment with the drug/PSA immediately before switching to the drug) for each treatment was analyzed using Pearson's correlation analysis. All statistical analyses were performed using IBM SPSS Statistics for Mac version 28.0 (IBM Japan, Tokyo, Japan). A value of < 0.05 was considered statistically significant.

## Results

**Fig. 1:**
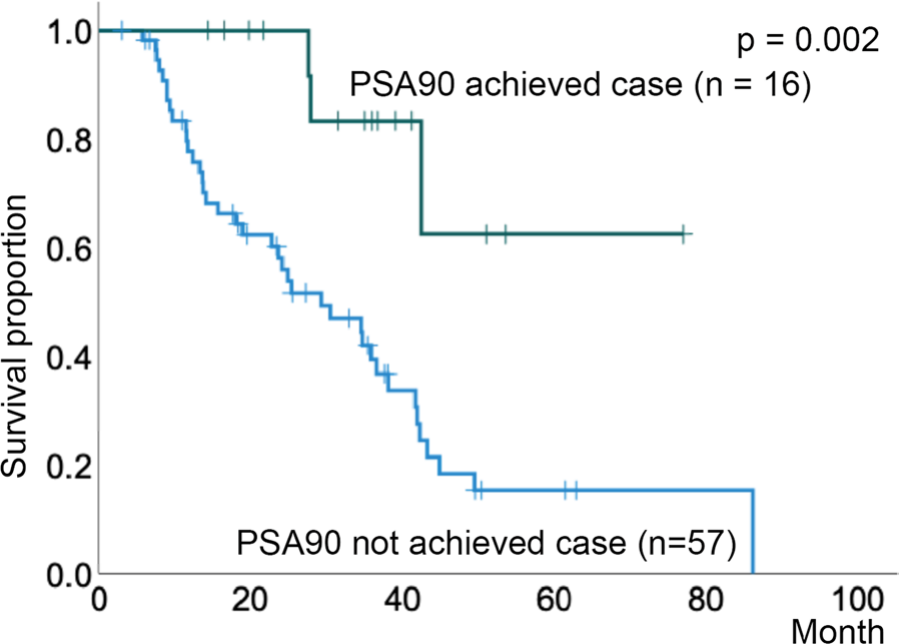
Kaplan–Meier estimates of overall survival after CRPC stratified by achievement of 90% PSA reduction (PSA90) after treatment with enzalutamide

**Fig. 2:**
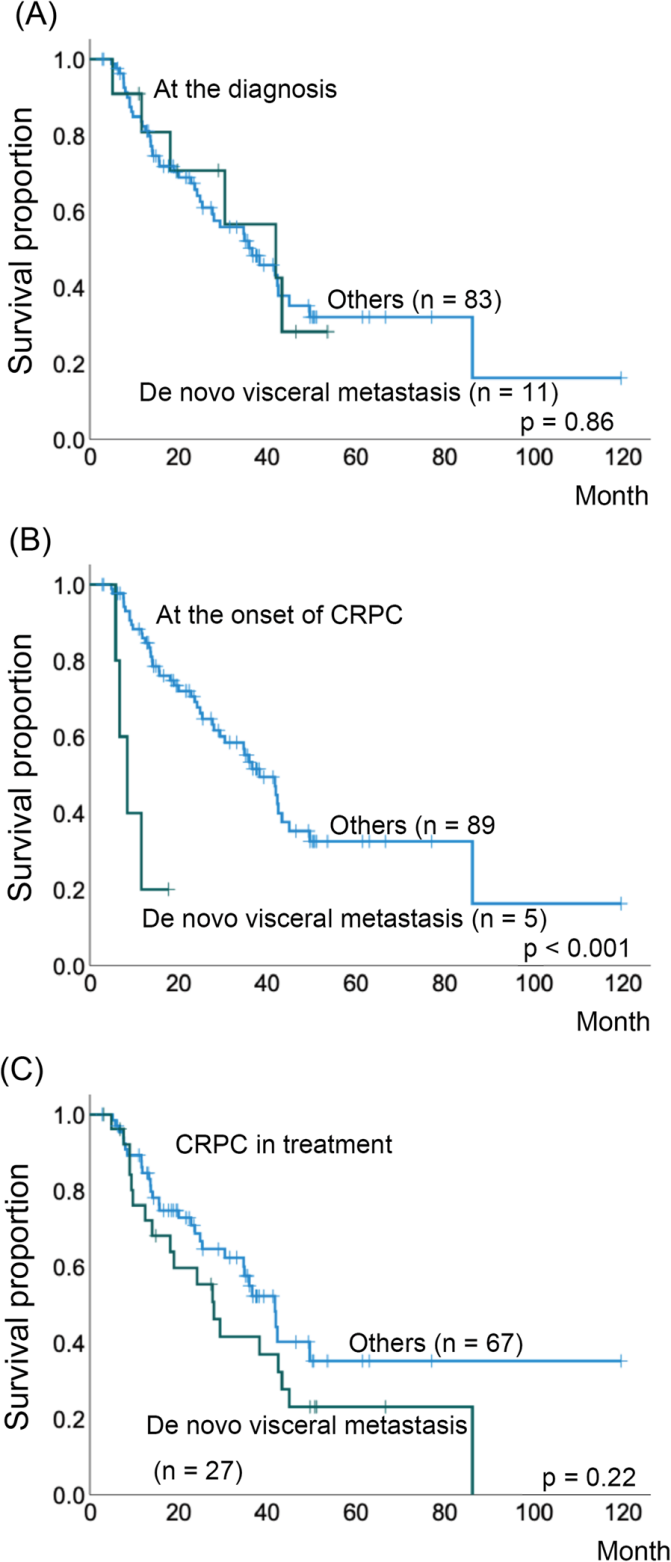
Kaplan–Meier estimates of overall survival after CRPC stratified by de novo visceral metastasis found at **A** diagnosis, **B** onset of CRPC, and **C** during treatment after CRPC diagnosis

The validity of the sample size required for the t-test was evaluated using G*power [9]. Setting the effect size to 0.5, alpha error to 0.08, power to 0.95, and two-sided test, the required sample size was 94. The median age and initial PSA level at the time of CRPC diagnosis was 76.0 years and 146.0 ng/ml, respectively (Table 1). The treatment sequence is summarized in Additional file 1: Fig. S1. Sixty-seven of the 94 patients had no metastases at the time of initial diagnosis (Table 1). Multivariate linear regression analysis showed that the time to CRPC duration was significantly correlated only with the duration of enzalutamide treatment (Table 2, p = 0.003). The patients who achieved 90% reduction in PSA levels (PSA90) from enzalutamide showed significantly better prognosis than those who did not (p = 0.002, Fig. 1). Comparison of prognosis according to the time of appearance of visceral metastases showed that patients with visceral metastases occurring at the time of CRPC diagnosis after bicalutamide or flutamide treatment had a significantly poorer prognosis (visceral metastases diagnosed at the prostate cancer diagnosis vs. others, p = 0.86; newly found at the onset of CPRC vs. others, p < 0.001; and newly found during CRPC treatment vs. others, p = 0.22, Fig. 2A–C).

**Table 1:** Patient characteristics (n = 94)

Characteristic	Value
Median (range) age at CRPC diagnosis (years)	76.0 (49–94)
Median (range) initial PSA level (ng/mL)	146.0 (4.10–10,672)
TNM classification at the diagnosis of prostate cancer	
T1	4
T2	17
T3	45
T4	28
N1	50
M1	67
Gleason score at the diagnosis of prostate cancer	
6	2
7	4
8	15
9	60
10	10
Unknown	3

*CRPC* castration-resistant prostate cancer, *PSA* prostate-specific antigen; *SD* standard deviation

**Table 2:** Multivariate linear regression analysis for model prediction

Model	Unstandardised coefficient		Standardised coefficient	t	Significance	95% CI for Β	
	B	SE	β			Lower bound	Upper bound
(Constant)	22.373	7.874		2.841	0.009	6.122	38.624
Enzalutamide treatment (months)	2.265	0.678	0.596	3.343	0.003	0.867	3.664
Docetaxel treatment (months)	− 0.688	0.523	− 0.231	− 1.314	0.201	− 1.768	0.392
Abiraterone treatment (months)	− 0.344	0.603	− 0.098	− 0.570	0.574	− 1.589	0.901

*CI* confidence interval, *SE* standard error

**Fig. 3:**
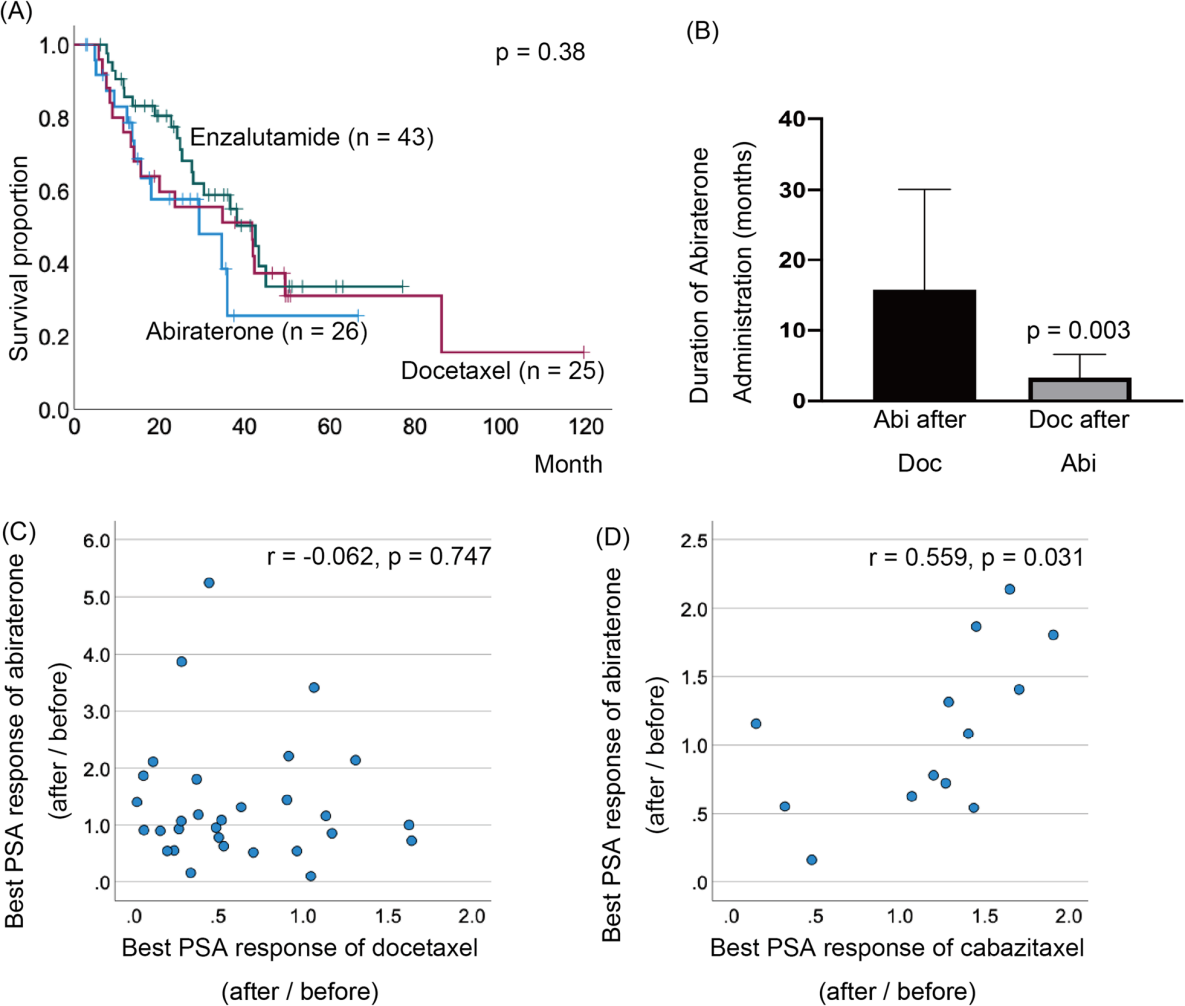
**A** Kaplan–Meier estimates of overall survival after CRPC with first-line CRPC treatments. **B** The duration of abiraterone treatment is compared according to the timing of its use (before and after docetaxel). *p < 0.05, Student’s t-tests. Bars, SD. Correlation of best PSA response to abiraterone with docetaxel (**C**) and cabazitaxel (**D**)

With respect to clinical treatment sequences, there was no difference in patient background except age at CRPC diagnosis according to the primary drugs used after CRPC diagnosis (Table 3). Furthermore, OS did not significantly differ according to the first-line drug treatment after CRPC (enzalutamide vs. abiraterone vs. docetaxel, p = 0.38, Fig. 3A). With respect to treatment sequences in CRPC, among patients who received both abiraterone and docetaxel, those who received docetaxel first had a significantly longer duration of abiraterone use than did those who received abiraterone first (docetaxel first vs. abiraterone first, p = 0.003, Fig. 3B). The time course of a representative case is shown in Additional file 2: Fig. S2. Although there was no correlation between docetaxel and abiraterone treatment with respect to the best PSA response, there was a positive correlation between abiraterone and cabazitaxel (abiraterone and docetaxel, p = 0.747; abiraterone and cabazitaxel, p = 0.031, Fig. 3C, D). On the other hand, regarding the best CRP and ALP response, neither abiraterone and docetaxel nor abiraterone and cabazitaxel showed a correlation (Additional file 3: Fig. S3).

**Table 3:** Comparison of patient characteristics by first-line therapies

	Abi (n = 26)	Enz (n = 43)	Doce (n = 25)	p-value
Mean (SD) age at CRPC diagnosis (years)	78.8 (8.8)*	77.8 (8.3)*	69.0 (7.5)	< 0.001
Initial PSA	1313.2 (2561.2)	533.1 (1653.2)	713.3 (1033.5)	0.242
TNM classification at the diagnosis of prostate cancer				
T1	0	3	1	0.65
T2	4	8	5	
T3	11	21	13	
T4	11	11	6	
N1 or higher	13	23	14	0.91
M1	20	30	17	0.74
Gleason scores				0.63
6	0	2	0	
7	1	1	2	
8	2	9	4	
9	20	24	16	
10	2	5	3	
Unknown	1	2	0	

*Abi* abiraterone, *CRPC* castration-resistant prostate cancer; *Doce* docetaxel, *Enz* enzalutamide, *PSA* prostate-specific antigen; *SD* standard deviation

*p < 0.05, vs. Group Doce by Turkey-Kramer test

## Discussion

Although newer agents have been recommended for the initial treatment of metastatic prostate cancer, bicalutamide and flutamide are still used to treat a large proportion of metastatic prostate cancer cases despite their risk of CRPC. This study found that subsequent enzalutamide therapy was associated with better prognosis in patients with a prolonged time to CRPC and initially treated with CAB. In addition, patients with a good PSA response to enzalutamide had a better prognosis. Furthermore, the prognosis varied greatly depending on the time of emergence of visceral metastases, and there was a correlation between abiraterone- and taxane-based chemotherapy with respect to treatment sensitivity.

The results of many randomized controlled trials and meta-analyses comparing CAB with ADT alone for prostate cancer are contradictory [10]. This may be due to the insufficient anti-androgenic effects of NSA. However, studies on the efficacy and safety of bicalutamide in Japanese prostate cancer patients have suggested that treatment with bicalutamide significantly prolongs survival without compromising patient safety or quality of life [11]. Therefore, CAB was recommended in Japan until the recent approval of second-generation antiandrogens for hormone-sensitive metastatic prostate cancer, and many Japanese patients with castration-resistant prostate cancer have been previously treated with this agent. Previously, the duration of prior response to ADT was reported to be a predictor of sensitivity to second-generation anti-androgens in CRPC patients [12]. Similarly, the present study found that the duration of response to CAB was related to the efficacy of enzalutamide. A recent study showed that AR alterations, including putative resistance mechanisms such as ligand-binding domain missense mutations, *AR* gene and/or upstream enhancer amplifications, and ligand-binding domain-truncating structural variants, were induced by clinical suppression of AR signaling [13]. One of these mechanisms is that incomplete androgen suppression leaves sufficient androgens to stimulate AR activity. In contrast, the negative feedback mechanism is not activated, resulting in increased expression of AR response genes that contribute to cell proliferation [14]. These results indicate that cases with a longer time to CRPC may not have *AR* mutations that are resistant to antiandrogens, and enzalutamide may be a good first choice for CRPC patients with long-term CAB.

Furthermore, spliceosomal protein production is enhanced in CRPCs, promoting AR splicing and expression [15]. In contrast, second-generation antiandrogens have been reported to promote genetic abnormalities in CRPC cells, leading to increased AR activity and the conversion of cancer cells to neuroendocrine tumors [13, 16]. In the present study, patients who developed visceral metastasis at the time of CRPC diagnosis had the worst prognosis, suggesting that more aggressive mutations than those induced by second-generation antiandrogens might likely have occurred in some of the CAB-treated patients.

Interestingly, the duration of abiraterone treatment was significantly longer when abiraterone was given after docetaxel treatment than when it was given before docetaxel treatment. There was a positive correlation between the efficacy of abiraterone and cabazitaxel. The CARD trial suggested that docetaxel followed by cabazitaxel is the best treatment sequence for metastatic CRPC patients who are refractory to second-generation AR inhibitors [17]. Meanwhile, switching between docetaxel/cabazitaxel and second-generation AR inhibitors has been reported to reverse gene mutation [18]. These data suggest that docetaxel should be the primary treatment of choice in CAB-treated patients with a short time to CRPC, and abiraterone should be administered before cabazitaxel if treatment is switched to docetaxel. In addition to the best PSA response, the best ALP and CRP responses were evaluated in this study. Neither the best CRP nor the best ALP response showed a correlation between abiraterone and taxanes as seen in the best PSA response. ALP has been suggested as a prognostic marker in prostate cancer [19]. On the other hand, ALP reflects bone turnover, osteoblast activity, and bone quality in adjacent bone tissue and may be particularly useful in combination with bone scintigraphy [19], which may be beneficial only in cases of bone metastases. In patients with CRPC receiving chemotherapy, elevated CRP is an independent predictor of overall and progression-free survival, suggesting its usefulness in combination with factors such as age, PSA, and serum alkaline phosphatase to predict chemotherapy response [20]. However, these studies evaluated CRP and ALP using baseline blood samples and did not examine variable values. Furthermore, this study included cases without bone metastases when they were treated with abiraterone or taxanes. This study had several limitations. First, this was a retrospective study in a single institution. The number of patients was small, and only Asian patients were evaluated. To address the study limitations, international multicenter studies are recommended in the future.

## Conclusion

This study presents the clinical characteristics of CRPC after CAB treatment.

Patients with a longer time to CRPC were more sensitive to enzalutamide, whereas those with CAB-induced visceral metastases had poorer prognosis. Abiraterone between docetaxel and cabazitaxel has a favorable therapeutic effect. These findings provide instrumental evidence that can enable better treatment selection for prostate cancer patients.

## Supplementary Information

Additional file 1. Fig. S1: Therapeutic drug sequencing is shown. Each drug was switched to the next at the discretion of the attending physician based on PSA elevation, progression on imaging, and side effects.

Additional file 2. Fig. S2: Clinical course of a representative case treated with docetaxel, abiraterone, and cabazitaxel. A 54-year-old man was referred for a prostate biopsy due to a high PSA level. Prostate cancer with a Gleason score of 4 + 4 + = 8 was diagnosed by prostate biopsy. Imaging studies showed bone and multiple lymph node metastases. After treatment with CAB, including bicalutamide and flutamide, he developed CRPC and was treated with docetaxel. However, the patient had side effects from docetaxel and was switched to abiraterone. Due to PSA rising on abiraterone, he was switched to cabazitaxel, and PSA has been slowly declining since then. During the disease course, lymph node metastases continued to shrink, and bone metastases did not worsen on imaging.

Additional file 3. Fig. S3: Correlation of the best ALP and CRP response to abiraterone with docetaxel and cabazitaxel.

## Data Availability

The data that support the findings of this study are available from the corresponding author, upon reasonable request.

## Abbreviations

AR: Androgen receptor
ADT: Androgen deprivation therapy
CAB: Combined androgen blockade
CRCP: Castration-resistant prostate cancer
NSA: Nonsteroidal androgen receptor antagonists
OS: Overall survival
PSA: Prostate-specific antigen
SD: Standard deviation
PSA90: 90% Reduction in PSA level
