# Supplementary material for: Clinical characteristics of patients with metastatic castration-resistant prostate cancer after treatment with combined androgen blockade
Source: BMC Urol. 2023 Apr 28;23:74. doi: 10.1186/s12894-023-01233-6 (PMC10148407; doi:10.1186/s12894-023-01233-6)
Supplement: Supplementary file 1 — Additional file 1. Fig. S1: Therapeutic drug sequencing is shown. Each drug was switched to the next at the discretion of the attending physician based on PSA elevation, progression on imaging, and side effects. [file 12894_2023_1233_MOESM1_ESM.docx]

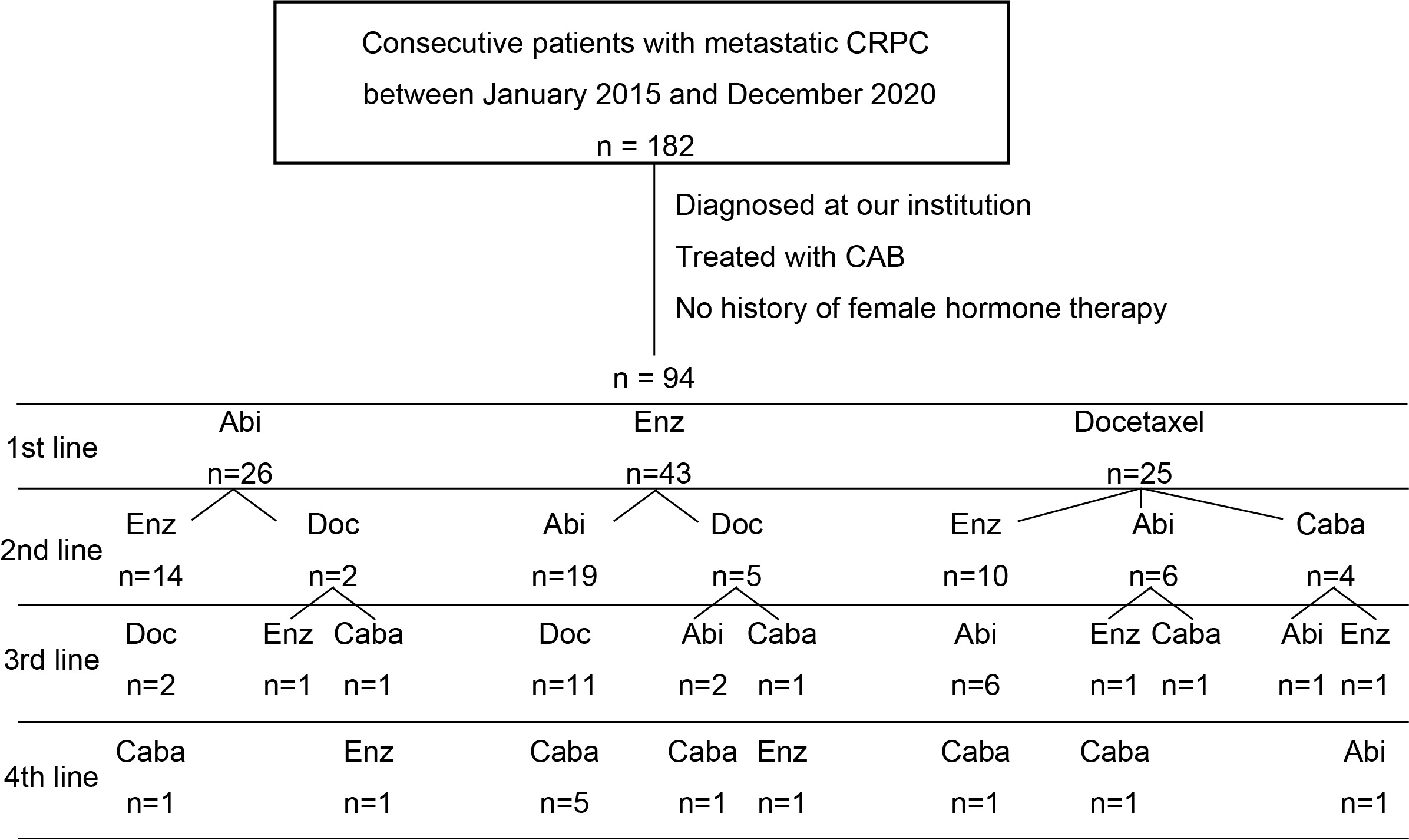


**Additional file 1: Fig. S1**

Therapeutic drug sequencing is shown. Each drug was switched to the next at the discretion of the attending physician based on PSA elevation, progression on imaging, and side effects.
