# Supplementary material for: Clinical characteristics of patients with metastatic castration-resistant prostate cancer after treatment with combined androgen blockade
Source: BMC Urol. 2023 Apr 28;23:74. doi: 10.1186/s12894-023-01233-6 (PMC10148407; doi:10.1186/s12894-023-01233-6)
Supplement: Supplementary file 2 — Additional file 2. Fig. S2: Clinical course of a representative case treated with docetaxel, abiraterone, and cabazitaxel. A 54-year-old man was referred for a prostate biopsy due to a high PSA level. Prostate cancer with a Gleason score of 4 + 4 + = 8 was diagnosed by prostate biopsy. Imaging studies showed bone and multiple lymph node metastases. After treatment with CAB, including bicalutamide and flutamide, he developed CRPC and was treated with docetaxel. However, the patient had side effects from docetaxel and was switched to abiraterone. Due to PSA rising on abiraterone, he was switched to cabazitaxel, and PSA has been slowly declining since then. During the disease course, lymph node metastases continued to shrink, and bone metastases did not worsen on imaging. [file 12894_2023_1233_MOESM2_ESM.docx]

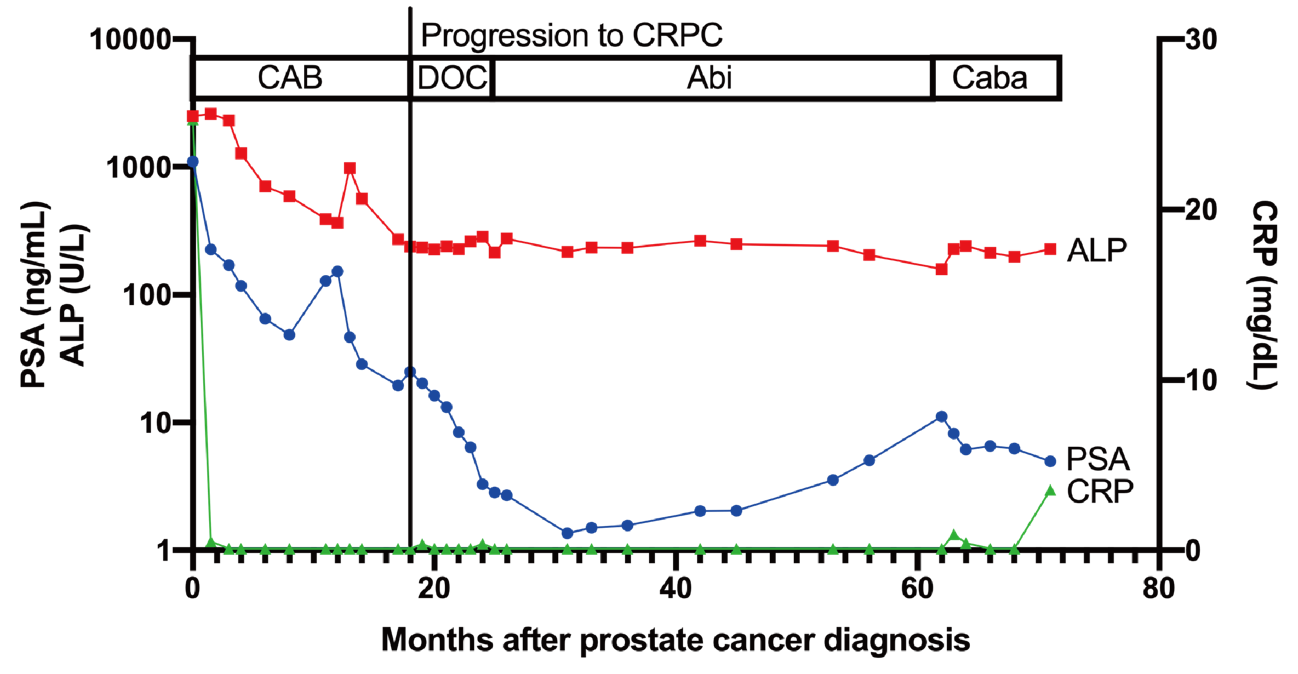


**Additional file 2: Fig. S2**

Clinical course of a representative case treated with docetaxel, abiraterone, and cabazitaxel. A 54-year-old man was referred for a prostate biopsy due to a high PSA level. Prostate cancer with a Gleason score of 4 + 4 = 8 was diagnosed by prostate biopsy. Imaging studies showed bone and multiple lymph node metastases. After treatment with CAB, including bicalutamide and flutamide, he developed CRPC and was treated with docetaxel. However, the patient had side effects from docetaxel and was switched to abiraterone. Due to PSA rising on abiraterone, he was switched to cabazitaxel, and PSA has been slowly declining since then. During the disease course, lymph node metastases continued to shrink, and bone metastases did not worsen on imaging.
