# Supplementary material for: Clinical characteristics of patients with metastatic castration-resistant prostate cancer after treatment with combined androgen blockade
Source: BMC Urol. 2023 Apr 28;23:74. doi: 10.1186/s12894-023-01233-6 (PMC10148407; doi:10.1186/s12894-023-01233-6)
Supplement: Supplementary file 3 — Additional file 3. Fig. S3: Correlation of the best ALP and CRP response to abiraterone with docetaxel and cabazitaxel. [file 12894_2023_1233_MOESM3_ESM.docx]

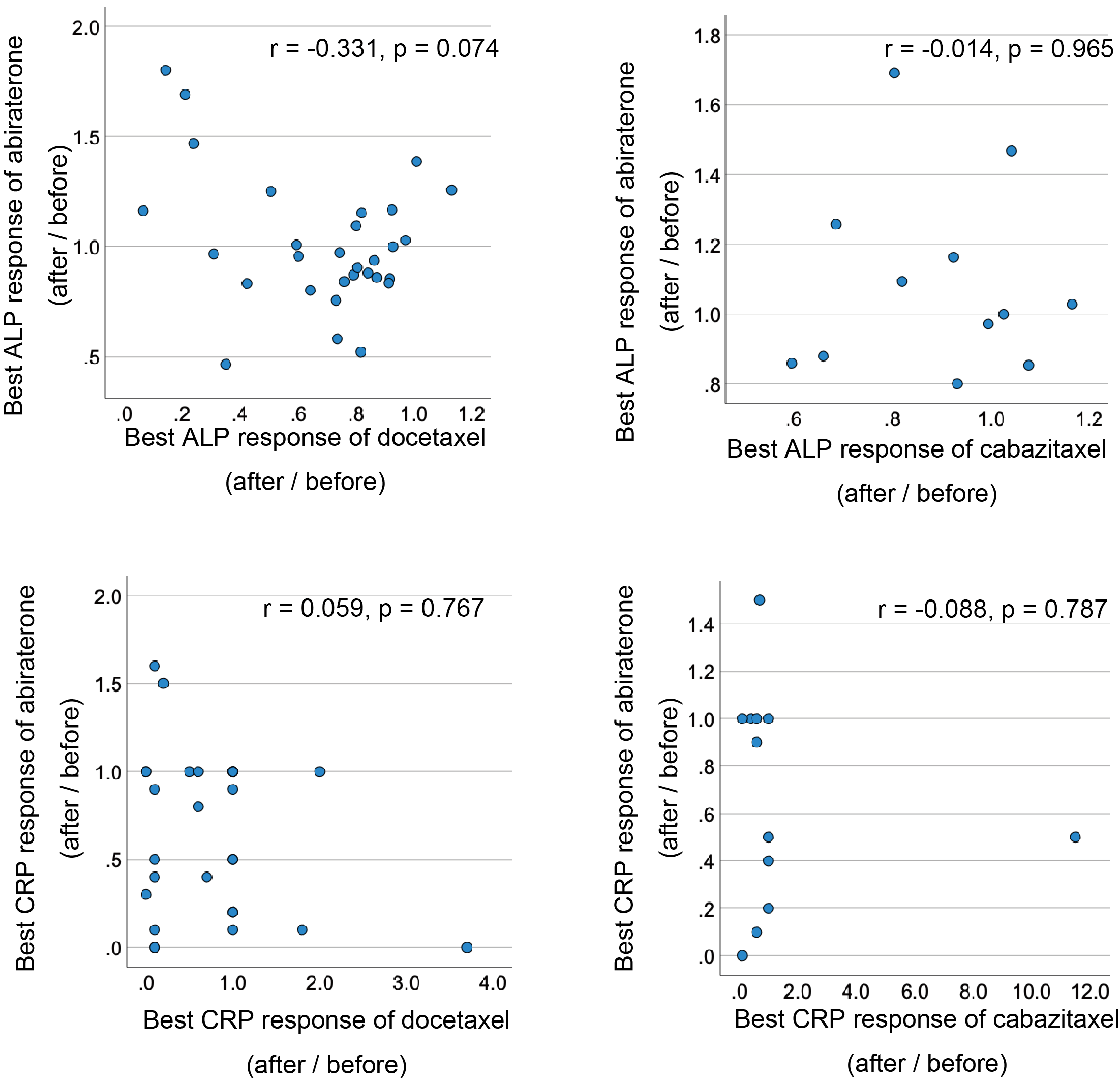


**Additional file 3: Fig. S3**

Correlation of the best ALP and CRP response to abiraterone with docetaxel and cabazitaxel.
